# Adaptive Atmospheric Light Estimation for Dehazing via a Novel Decoupled Scattering Model with Neutral-Pixel and Visual-Depth Priors

**DOI:** 10.3390/jimaging12050218

**Published:** 2026-05-21

**Authors:** Zhu Zhu, Xiaoguo Zhang

**Affiliations:** 1 School of Computer Engineering, Jinling Institute of Technology, Nanjing 211169, China; edileon@jit.edu.cn; 2 School of Instrument Science and Engineering, Southeast University, Nanjing 210096, China

**Keywords:** atmospheric light, dehazing, color casts, exposure

## Abstract

Accurate estimation of atmospheric light (AL) is essential within the atmospheric scattering model (ASM) to achieve high-quality image dehazing. Most existing methods, however, typically assume spatial uniformity of AL and rely on heuristic estimation from distant pixels, which often results in color distortion and exposure imbalance in dehazed outputs. To address this issue, we propose a novel framework that decouples AL into distinct color and intensity components. Specifically, a neutral pixel prior (NPP) is introduced for precise AL color estimation, which can eliminate color casts. For AL intensity estimation, an adaptive global-local fusion strategy integrating luminance perception transformation and a depth-related color prior (DRCP) is developed to realize balanced exposure. Extensive experiments demonstrate that the proposed method significantly outperforms state-of-the-art AL estimation methods, yielding dehazed images with enhanced color fidelity and more natural illumination.

## 1. Introduction

The atmospheric scattering model (ASM) is the mainstream framework in image dehazing [1,2,3,4,5,6,7,8,9,10,11,12,13,14,15,16,17,18,19,20,21]. It describes a hazy image as:

(1) I(x)=t(x)J(x)+A(1−t(x))

where ***I*** denotes the observed hazy image, *x* is a pixel coordinate, ***J*** represents the haze-free scene radiance, ***A*** is the atmospheric light (AL), and *t* is the transmission map.

**Figure 1:**
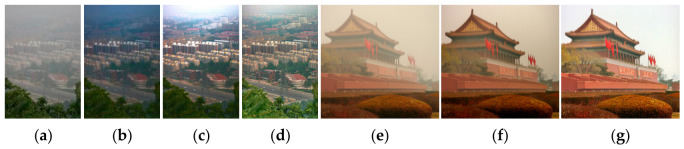
Critical role of AL estimation on ASM-based dehazing: (**a**,**e**) hazy images; (**b**) Under-exposure result; (**c**) Over-exposure result; (**f**) residual color casts; (**d**,**g**) our results.

In ASM-based dehazing, significant attention has been paid to transmission estimation, resulting in various methods such as the dark channel prior (DCP) [4], the ambient light similarity prior (ALSP) [5], the haze-line (HL) [6], the rank-one prior (ROP) [12], and multi-scale convolutional networks (MSCNN) [15]. In contrast, AL estimation has received less attention but plays a critical role in dehazing. Specifically, accurate AL estimation ensures color fidelity and balanced exposure in dehazed results (Figure 1d,g). Conversely, an inaccurate AL often leads to imbalanced exposure or residual color casts in dehazed outputs (Figure 1b,c,f).

Despite their diverse strategies, existing AL estimation methods share a common, intrinsic limitation: the entangled recovery of color and intensity components predicated on the assumption of global uniformity. This constraint is most evident in early heuristics that directly inferred AL from primitive pixel statistics—such as selecting the brightest intensities [17,18], assuming a canonical white light [19], or employing per-channel maxima [20]. Such simplistic paradigms inherently struggle to distinguish between the ambient illumination and the intrinsic reflectance of scene objects. Consequently, these methods are prone to misinterpreting bright foreground objects as infinite-depth AL sources. This misalignment in AL localization typically results in an overestimation of the atmospheric light magnitude, yielding dehazed results characterized by severe underexposure and a persistent loss of visual contrast.

To address the limitations of simple statistical heuristics, subsequent research has pivoted toward leveraging sophisticated scene priors to enhance the robustness of AL estimation. For instance, He et al. [4] introduced the dark channel prior (DCP) to estimate AL by identifying pixels in non-sky regions with near-zero intensity in at least one color channel. Berman et al. [5] formulated the non-local haze-line model, characterizing pixel distributions in RGB space as lines that converge at the air-light coordinate to locate infinite-depth pixels. Liu et al. [12] proposed the rank-one prior (ROP), which derives AL by exploiting the low-rank structural correlation between the transmission map and the degraded image. Meanwhile, Gautam et al. [21] applied a color constancy prior (CCP) to effectively distinguish dense-haze regions from white objects by analyzing spectral consistency.

Beyond these prior-based approaches, alternative methodologies have focused on structural recurrence and recursive search. Bahat et al. [22] utilized internal patch recurrence (IPR) to estimate the ambient light by matching recurring patches across different depth layers. Complementarily, Ju et al. [9] proposed a quad-tree recursive strategy that adaptively locates AL candidates guided by a specialized haze density estimation model. While these advanced strategies achieve more precise AL localization, they still operate under the assumption of global uniformity and rely on a coupled estimation mechanism. Consequently, the restored results often suffer from residual color casts and exposure imbalances, particularly in complex, non-uniform illumination environments.

We also note recent advances in data-efficient training [23] and latent graph attention [24] for general visual tasks; however, they are orthogonal to our physical-prior dehazing approach and are not directly compared.

In summary, existing methods are hampered by two fundamental limitations: the oversimplified global uniformity assumption, which overlooks natural illumination variations, and the entangled recovery mechanism that fails to treat color (spectral casts) and intensity (local adaptation) as distinct physical entities.

To address these challenges, we propose a comprehensive framework with three core contributions:

- Physical Model Reformulation: We decouple atmospheric light (AL) into independent color and intensity components, leading to a refined atmospheric scattering model (ASM) that better aligns with physical reality.
- Neutral Pixel Prior (NPP): A novel prior that leverages the intrinsic color consistency between AL and neutral pixels, enabling robust and accurate spectral cast estimation.
- Dual-Path Intensity Estimation: An adaptive global-local AL intensity estimation method that synergistically fuses luminance perception transformation (LPT) and a depth-related color prior (DRCP) to achieve optimal exposure balance and mitigate artifacts.

Extensive experiments validate the superior accuracy of our AL estimation, which consistently yields dehazed results with enhanced visual quality and structural fidelity. The remainder of this paper is organized as follows. Section 2 provides a comprehensive review of related work concerning atmospheric light estimation and image dehazing. Section 3 details the proposed decoupled atmospheric scattering model, including the neutral pixel prior (NPP) and the adaptive fusion mechanism. In Section 4, we present extensive experimental results and comparative analyses to evaluate the performance of our method. Finally, Section 5 concludes the paper and discusses potential directions for future research.

## 2. Proposed Methodology

To overcome the inherent constraints of conventional holistic estimation paradigms, we propose a physically decoupled framework that independently addresses the spectral and radiant attributes of atmospheric illumination. The core philosophy of our methodology is to reformulate the classical atmospheric scattering model (ASM) into a bifurcated structure, treating the spectral composition (color) and radiant flux (intensity) as distinct physical entities. Specifically, unlike traditional approaches that represent atmospheric light (AL) as a monolithic vector, our model decomposes it into a unit color vector (H) and a spatially adaptive intensity scalar (V). This decomposition enables more precise characterization of the haze effect, particularly in complex scenes where non-uniform illumination or significant chromatic shifts compromise standard global assumptions.

**Figure 2:**
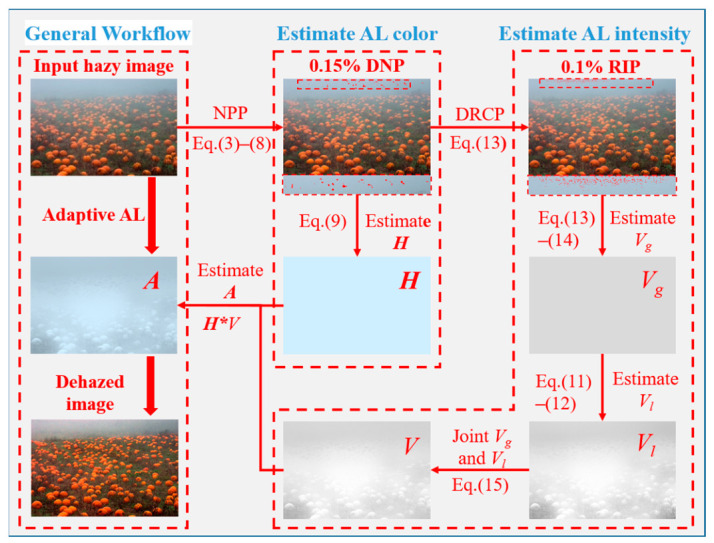
Flowchart of the proposed algorithm.

As illustrated in the conceptual pipeline (Figure 2), the estimation process is executed through two specialized, synergistic modules. First, the global AL color is recovered via a neutral pixel prior (NPP), which capitalizes on the intrinsic chromatic consistency between the atmospheric light and neutral scene elements. We posit that while neutral candidates are statistically abundant across natural scenes, their identification in perceptually uniform regions is frequently hindered by pervasive color casts. To resolve this, our framework identifies detectable neutral pixels (DNP) within textured regions characterized by local intensity variations. In these areas, the inter-channel variance provides a robust discriminative feature that remains resilient to spectral shifts, enabling a stable recovery of H by decoupling the neutral signal from the atmospheric interference.

Concurrently, to ensure balanced exposure across the recovered scene, we introduce an adaptive global-local fusion strategy for AL intensity estimation. This strategy integrates a depth-related color prior (DRCP) with a luminance perception transformation (LPT). The DRCP is employed to anchor the global baseline of the atmospheric light intensity by leveraging depth-dependent scene information, ensuring radiometric grounding in distant regions. Complementarily, the LPT facilitates localized intensity refinement by perceiving and adjusting to ambient lighting variations within the scene. The interplay between these two priors allows the framework to dynamically reconcile global luminance coherence with the need for fine-grained contrast enhancement in regions of varying haze density. By modularizing the estimation into these distinct components, the proposed methodology ensures a consistent scene recovery that adaptively addresses both global color distortion and local visibility degradation.

## 3. The Proposed Atmospheric Light Estimation Method

In this section, we detail the proposed framework designed to rectify the exposure imbalances and color distortions prevalent in existing dehazing results. Unlike prior arts that estimate AL as a monolithic vector, our approach advocates for a “divide-and-conquer” strategy: decoupling the AL into color (governing spectral consistency) and intensity (governing local adaptation). We first present the mathematical reformation of the ASM, followed by the derivation of the neutral pixel prior (NPP) for robust color estimation. Subsequently, we describe the physics-based fusion strategy that synergizes global coherence with local contrast fidelity. This hierarchical design ensures that the atmospheric light is not only accurately localized but also physically consistent with the heterogeneous illumination of natural scenes.

### 3.1. Atmospheric Light Decoupling and ASM Reformation

We decouple atmospheric light into color and intensity components based on their distinct physical roles in image formation. The color component, influenced by scattering particles and light sources, captures the spectral properties of ambient illumination and induces color casts. Conversely, the intensity component, which varies spatially, corresponds to the radiant flux and determines scene brightness. This physical distinction leads to a decoupled framework, allowing color and intensity to be processed separately for color fidelity and exposure balance.

Based on this rationale, we reformulate ASM by decoupling AL into a normalized color vector ***H*** (${\left\|H\right\|}_{\infty }=1$) and a scalar intensity V(x).

(2) I(x)=t(x)J(x)·H+V(x)(1−t(x))H

where the color vector H and scalar intensity V(x) are used to eliminate color casts and resolve exposure imbalance in dehazing, respectively (Figure 1d,g).

Crucially, our model maintains backward compatibility: it reduces to the traditional ASM when the color vector is spectrally neutral (H=[1,1,1]) and the intensity is spatially constant. Thus, the traditional model represents a specific instance of our decoupled framework, defined by neutral scattering and uniform illumination.

### 3.2. Atmospheric Light Color Estimation via Neutral Pixel Prior

For a color-neutral scene point whose intrinsic radiance is achromatic, i.e., J(x)=J(x)·[1,1,1] with J(x) being its scalar intensity, substituting it into Equation (2) gives:

(3) I(x)=(t(x)J(x)+V(x)(1−t(x)))H=S(x)·H

where *S*(*x*) is a positive scalar. Consequently, the normalized color of ***I***(*x*) is independent of *S*(*x*) and directly equals the AL color.

(4) $$\frac{I\left(x\right)}{{\left\|I\left(x\right)\right\|}_{\infty }}=\frac{S\left(x\right)\cdot H}{{\left\|S\left(x\right)\cdot H\right\|}_{\infty }}=H$$

Equation (4) reveals a deterministic physical principle: for a neutral scene point, the color of its hazy observation is ***H***. It forms the foundation of our neutral pixel prior (NPP).

**Figure 3:**
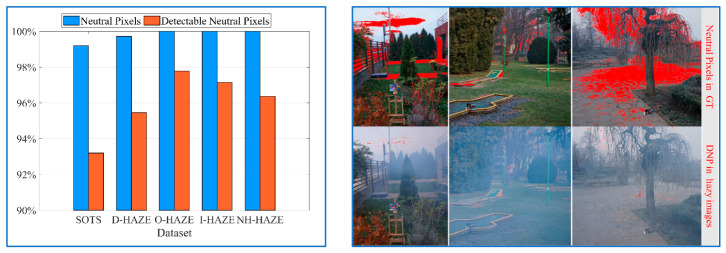
Proportion of images containing neutral pixels and detectable neutral pixels in five hazy datasets (**left**) and visual examples (**right**).

Neutral pixels are widespread in natural scenes, as confirmed by five standard hazy datasets (SOTS [25], D-HAZE [26], NH-HAZE [27], I-HAZE [28], O-HAZE [29]; see Figure 3). While these pixels are most common in perceptually uniform regions, their detection in these regions is hindered by color casts due to inherently low local variance. Conversely, detail regions often preserve detectable neutral pixels (DNP), where comparable inter-channel variance (Equations (5)–(8)) confers robustness against color casts, enabling reliable AL color estimation.

Given the prevalence of neutral pixels (Figure 3) and the above analysis, we define two essential criteria for DNP: (1) they must be in a small neutral region, and (2) the local variance within this region must be non-zero. Under these constraints, DNP can be identified in logarithmic space.

Taking the logarithm of both sides of Equation (2) for each color channel c∈{r,g,b}, and using the property that $log\left(H^{c}\right)$ is constant per channel, we obtain:

(5) $$log\left(I^{c}\left(x\right)\right)=log\left(H^{c}\right)+log\left(t\left(x\right)J^{c}\left(x\right)+V\left(x\right)\left(1-t\left(x\right)\right)\right)$$

Given that $H^{c}$ is assumed to be spatially uniform across the image, its contribution to the local variance is zero; therefore, the local variance on the right-hand side of Equation (5) depends solely on the second term, and we obtain:

(6) $${Var}_{\eta }\left(log\left(I^{c}\left(x\right)\right)\right)={Var}_{\eta }\left(log\left(t\left(x\right)J^{c}\left(x\right)+V\left(x\right)\left(1-t\left(x\right)\right)\right)\right)$$

where ${Var}_{\eta }\left(\cdot \right)$ computes, for each color channel independently, the sample variance of the pixel values within a η×η local window centered at *x* in the log domain. To enforce strict local consistency, we set *η* to a small, compact window size, ensuring that both t(x) and V(x) can be considered approximately uniform within this local region. Given criteria (1) and (2) for a neutral pixel, the term $t\left(x\right)J^{c}\left(x\right)+V\left(x\right)\left(1-t\left(x\right)\right)$ is equal for each channel *c*. Consequently, the three-channel local variances in Equation (6) are equal. This leads to equal variances for the corresponding observed pixel values on the left-hand side. It demonstrates that the neutrality of a pixel can be quantified by evaluating the consistency of local variances in the logarithmic domain. Accordingly, we define a neutrality index P(x) as follows. Let ${Vr}_{\eta }^{c}\left(x\right)={Var}_{\eta }\left(log\left(I^{c}\left(x\right)\right)\right)$ for each channel *c*. Then,

(7) $$P\left(x\right)=\sqrt{\frac{1}{3}\sum_{c\in \left\{r,g,b\right\}}\frac{{\left({Vr}_{\eta }^{c}\left(x\right)-{\bar{Vr}}_{\eta }\left(x\right)\right)}^{2}}{{{\bar{Vr}}_{\eta }\left(x\right)}^{2}}}$$

where ${\bar{Vr}}_{\eta }\left(x\right)$ is the mean variance over the three channels, and P(x) measures the color neutrality of pixel x. The division by ${{\bar{Vr}}_{\eta }\left(x\right)}^{2}$ makes the index scale-invariant, i.e., it depends only on the relative differences among the three channel variances, not on their absolute magnitudes. A smaller P(x) indicates a more neutral pixel. Perfect neutrality is approached when (1) P(x)→0 and (2) the three channel variances are equal and non-zero, i.e., ${Vr}_{\eta }^{r}\left(x\right)={Vr}_{\eta }^{g}\left(x\right)={Vr}_{\eta }^{b}\left(x\right)\neq 0$. The requirement of non-zero variance serves to exclude smooth textureless regions, where the variances are near zero and would otherwise yield misleadingly small *P*(*x*) values due to chance equality, even if the scene radiance is not neutral. If ${Vr}_{\eta }^{c}\left(x\right)<\epsilon$ (e.g., $\epsilon =10^{-4}$) for every channel, we regard the pixel as belonging to a textureless region and set its *P*(*x*) to the global maximum of *P* to avoid numerical instability and false detections. This criterion also excludes textureless white objects, as their local variances are near zero in all three channels.

To mitigate the interference from low-luminosity pixels, we define the refined neutrality index as:

(8) $$\bar{P}\left(x\right)={boxfilter}_{\eta_{1}}\left(\frac{P\left(x\right)}{Q\left(x\right)}\right)$$

where ${boxfilter}_{\eta_{1}}\left(\cdot \right)$ denotes a $\eta_{1}\times \eta_{1}$ local averaging filter, and Q(x) is the per-pixel mean intensity of the three color channels. The division by Q(x) down-weights the influence of dark pixels because their low intensity would otherwise amplify unstable variance ratios. The filtered response map is then min-max normalized to [0, 1], yielding the final neutrality index. A smaller $\bar{P}\left(x\right)$ indicates a more reliable neutral pixel. Therefore, by selecting the pixel with the smallest $\bar{P}\left(x\right)$ values, we can estimate the atmospheric light color more accurately.

**Figure 4:**
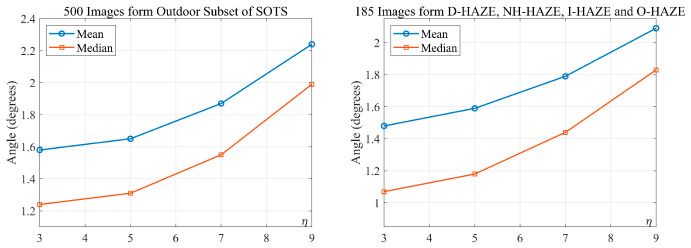
Angular error between estimated and ground-truth AL color under different variance window sizes (no smoothing). The 3 × 3 window yields the smallest error because larger windows may include non-neutral neighbors.

**Figure 5:**
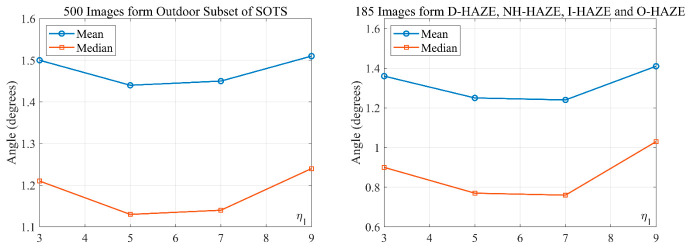
Angular error after smoothing with different kernel sizes (fixed 3 × 3 variance window).

To determine appropriate window sizes for variance estimation and smoothing, we first evaluated four variance window sizes (3 × 3, 5 × 5, 7 × 7, and 9 × 9) without any smoothing, using the angular error between the estimated H and the ground-truth color as the metric. As shown in Figure 4, the 3 × 3 yields the smallest error because larger windows tend to incorporate non-neutral pixels from neighboring regions, which is particularly problematic when neutral pixels are scarce. With the optimal variance window fixed at 3 × 3 (η=3), we then tested smoothing kernels of sizes 3 × 3, 5 × 5, 7 × 7, and 9 × 9. Figure 5 shows that the 5 × 5 and 7 × 7 kernels yield the lowest angular errors. For simplicity, we adopt the 5 × 5 filter ($\eta_{1}=5$). The 3 × 3 variance window preserves near-pixel-level neutral information, and the subsequent 5 × 5 smoothing reduces noise while following image structures.

As shown in Figure 3, detectable neutral pixels exist in the vast majority of hazy images across different datasets. According to Equation (8), we select the top *n*% of pixels with the smallest $\bar{P}\left(x\right)$ values as the candidate neutral set Ω and then estimate the atmospheric light color H via the NPP as follows:

(9) $$H={\left(\sum_{\left(x\right)\in \Omega }{I\left(x\right)}^{\sigma }\right)}^{\frac{1}{\sigma }}/{\left\|{\left(\sum_{\left(x\right)\in \Omega }{I\left(x\right)}^{\sigma }\right)}^{\frac{1}{\sigma }}\right\|}_{\infty }$$

where σ is the order of the Minkowski norm, set to 5 for optimal performance following [30].

**Figure 6:**
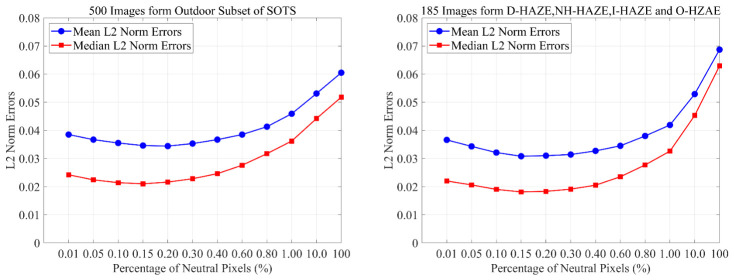
Determination of the optimal sampling percentage *n* based on the L2 error between the estimated and ground-truth atmospheric light color across five datasets.

To determine the optimal sampling rate *n*, we conducted a systematic validation on images from five datasets: SOTS (500 outdoor images), D-HAZE (55), NH-HAZE (55), I-HAZE (30), and O-HAZE (45). The ground-truth AL color was manually annotated for all these images. As shown in Figure 6, the L2-norm errors between the estimated H and the ground-truth reach their minimum at *n* = 0.15%. Accordingly, we select the top 0.15% of pixels with the smallest $\bar{P}\left(x\right)$ values for estimating H.

Dividing Equation (2) by ***H*** removes color cast in a hazy image, yielding:

(10) $$\overset{ˇ}{I}\left(x\right)=t\left(x\right)\cdot J\left(x\right)+V\left(x\right)\cdot \left(1-t\left(x\right)\right)$$

where $\overset{ˇ}{I}\left(x\right)=I\left(x\right)/H$ denotes the color-corrected image. Equation (10) retains the form of the traditional ASM (Equation (1)), while incorporating intensity adaptation through V(x).

### 3.3. Atmospheric Light Intensity Estimation via Luminance Perception Transformation and Depth-Related Color Prior

Adopting a spatially uniform assumption for atmospheric light intensity invariably leads to significant exposure artifacts. Specifically, overestimating this parameter results in global underexposure of the recovered scene, whereas underestimation causes irreversible clipping of local highlights (as illustrated in Figure 1b,c). This failure stems from the inherent spatial heterogeneity of natural illumination, which makes global exposure normalization, predicated on a monolithic constant, physically unattainable in complex environments.

To address these limitations, we propose a two-path estimation strategy that separately estimates the global and local AL intensities, denoted as $V_{g}$ and $V_{l}$, from the observed hazy image. In this framework, $V_{g}$ serves as a baseline constraint to control the overall exposure level of the image, while $V_{l}$ captures local exposure variations caused by non-uniform illumination. By dynamically fusing $V_{g}$ and $V_{l}$, we obtain a radiometrically balanced output: the dehazed image maintains global exposure consistency while adaptively preserving local contrast and structural details.

The estimation of local AL intensity is based on the luminance perception transformation (LPT). The core insight stems from the decoupled ASM in Equation (2): in a local region $\Omega_{l}\left(x\right)$, the AL intensity $V_{l}\left(x\right)$ constitutes the theoretical upper bound of pixel intensities in that region. This is based on a principle of physical optics: the scene radiance (e.g., $J^{c}\left(y\right)$, $y\in \Omega_{l}\left(x\right)$) cannot exceed the intensity of its illumination source, i.e., $J^{c}\left(y\right)\leq V_{l}\left(x\right)$. Since both $V_{l}$ and H are constant within the local region, substituting them into Equation (2) yields $I^{c}\left(y\right)\leq V_{l}\left(x\right)$ Therefore, the maximum pixel intensity in the local region provides an asymptotically unbiased estimate of $V_{l}\left(x\right)$. This estimation approaches optimality when the region contains pixels with very low transmission. To obtain this estimate, we define the luminance map *B*(*x*) via LPT as:

(11) $$B\left(x\right)=\underset{y\in \Omega_{l}\left(x\right)}{\max}\left(\underset{c\in \left\{r,g,b\right\}}{\max}\left(I^{c}\left(y\right)\right)\right)$$

where $\Omega_{l}\left(x\right)$ denotes a local region centered at *x* (with a radius set to 15 in our paper) [31]. We regularize B with a guided filter GF(·) [32] to suppress noise from the extremum operation and produce a smooth *V_l_* map that conforms to natural illumination.

(12) $$V_{l}\left(x\right)=GF\left(B\left(x\right)\right)$$

From Equation (2), the global AL intensity is conventionally interpreted as the atmospheric light at an infinite distance in the scene. However, real-world images rarely contain pixels that strictly satisfy this infinite-depth assumption. To address this issue, we introduce a method to estimate the global AL intensity by leveraging a visual depth perception mechanism.

**Figure 7:**
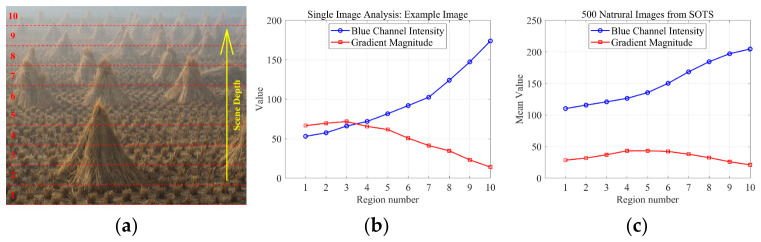
Variation of blue channel intensity and detail level with respect to scene depth. (**a**) Example image, (**b**) Variation of blue channel intensity and detail level with respect to scene depth, (**c**) Average trends of intensity and detail across 500 natural hazy images from SOTS.

It is observed in hazy images that the brightness of the blue channel exhibits a significant positive correlation with scene depth [11], while image detail is negatively correlated with depth. As visual depth increases, blue channel intensity tends to rise, and image details gradually diminish, as illustrated in Figure 7. Inspired by the color attenuation prior [8], which links scene depth to the difference between brightness and saturation, we propose a depth-related color prior (DRCP) and formulate a visual depth perception factor *D*(*x*) to identify relatively infinite-depth pixels (RIP) within the image

(13) $$D\left(x\right)=\frac{{\bar{Vr}}_{\eta }\left(x\right)}{I^{b}\left(x\right)+\epsilon }$$

where ${\bar{Vr}}_{\eta }\left(x\right)$ is the mean local variance across the three channels (as in Equation (7)), and ϵ is a small constant. A smaller *D*(*x*) indicates greater depth.

Following common strategies for infinite-depth pixel extraction [4,8,9,12], we select the top 0.1% of pixels with the smallest D(x) values as candidate RIP. Then, the global AL intensity $V_{g}$ is obtained as:

(14) $$V_{g}=\frac{1}{\left|RIP\right|}\sum_{x\in RIP}\underset{c\in \left\{r,g,b\right\}}{\max}\left(I^{c}\left(x\right)\right)$$

where RIP denotes the set of candidate pixels.

To physically integrate the global and local AL intensities, we propose an adaptive fusion mechanism inspired by the exponential decay of visibility in scattering media (the Beer–Lambert law). Specifically, the fused intensity V(x) is defined as a spatially varying weighted sum:

(15) $$\left(x\right)=\left(1-\omega \left(x\right)\right){\cdot V}_{g}+\omega \left(x\right){\cdot V}_{l}\left(x\right)$$

where the weighting factor ω(x) is given by

(16) $$\omega \left(x\right)=exp\left(-\frac{V_{l}\left(x\right)}{T}\right)$$

The scale parameter *T* is adaptively determined by applying Otsu’s thresholding to the $V_{l}$ map. Otsu’s method separates the $V_{l}$ map into two classes, near-field detail-rich regions and far-field haze-dominant regions, by maximizing the inter-class variance. The resulting threshold *T* provides a natural division. In bright regions where $V_{l}\left(x\right)\geq T$ (likely far-field or haze-dominant), ω(x) approaches zero. So, the fused intensity defaults to the global estimate $V_{g}$, ensuring long-range consistency. In darker regions where $V_{l}\left(x\right)<T$, (near-field, detail-rich), ω(x) tends to one, preserving local intensity variations from $V_{l}\left(x\right)$.

In the special case of nearly uniform haze, the $V_{l}$ map is approximately constant. Otsu’s threshold then lies near the mean intensity, making ω(x) nearly constant across the image. As a result, the fusion effectively reduces to using a global $V_{g}$ alone, which avoids introducing local artifacts or halos. Thus, the proposed fusion strategy requires no hand-tuned parameters and adapts to the content of each image.

**Figure 8:**
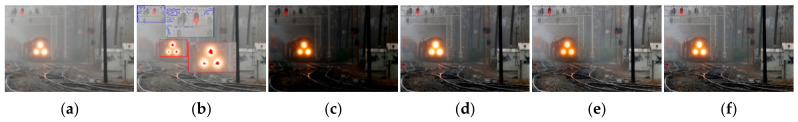
Ablation study on AL intensity estimation. (**a**) Input hazy image with localized intense light interference. (**b**) AL pixel localization (Red box: conventional; Blue box: DRCP). Dehazed results using: (**c**) conventional global AL intensity; (**d**) our global AL intensity; (**e**) our local AL intensity; (**f**) our fused AL intensity.

Figure 8 presents an ablation analysis of AL intensity under non-uniform illumination. The input hazy image (Figure 8a) contains a localized bright light source. Conventional methods tend to identify this local source as the global AL (red box in Figure 8b). This leads to severe global underexposure in the dehazed result (Figure 8c). By contrast, our DRCP locates a more appropriate global AL (blue box in Figure 8b). Dehazing with our global estimate ($V_{g}$) yields proper overall exposure but causes local underexposure in dark areas (Figure 8d). Similarly, using only the local AL ($V_{l}$) improves mid-tone balance but leads to highlight underexposure (Figure 8e). In comparison, the fused AL intensity (V) achieves both balanced global exposure and local illumination preservation (Figure 8f). These results validate the necessity of our global-local fusion strategy.

## 4. Experimental Results and Discussion

We evaluate our AL estimation framework on five public hazy datasets: SOTS (500 outdoor images), D-HAZE (55), I-HAZE (30), NH-HAZE (55), and O-HAZE (45). Additionally, a custom set of 35 natural hazy images with manually annotated ground-truth AL is used to assess the standalone AL estimation accuracy. Both the standalone AL estimation accuracy and the downstream improvement on existing dehazing algorithms are assessed. All hyperparameters are set as described in Section 3 and are kept unchanged across all experiments. Baseline methods use their default settings. No dataset-specific tuning is applied, ensuring a fair comparison.

**Figure 9:**
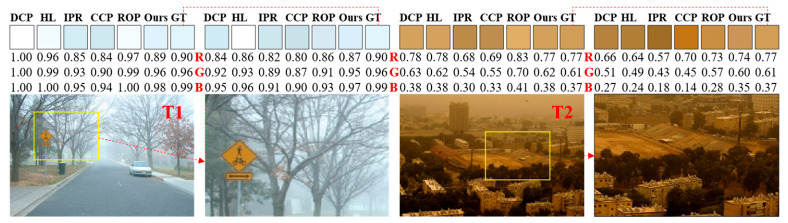
Qualitative Comparison of Atmospheric Light Estimation on Two Hazy Images.

**Figure 10:**
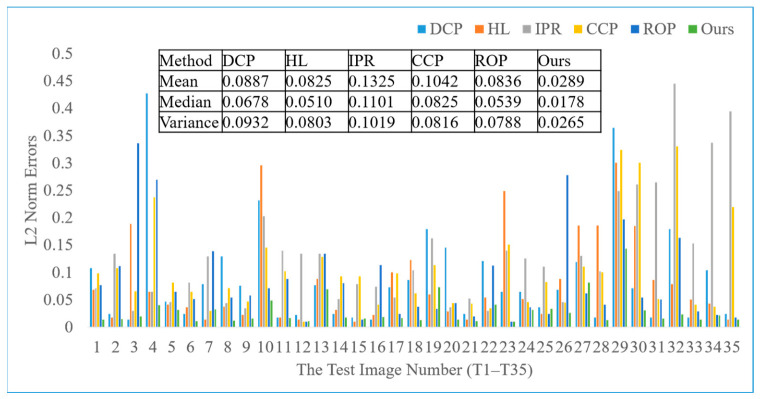
Quantitative Evaluation of AL Estimation Accuracy: L2-norm Errors on 35 Hazy Images.

To demonstrate the methodological advantages of our decoupled approach, we performed a comparative analysis against five mainstream baseline algorithms: DCP [4], HL [6], IPR [22], ROP [12], and CCP [21]. This evaluation was carried out on a curated dataset comprising 35 natural hazy images, for which ground-truth AL values were manually annotated to provide a reliable reference (Ground-truth AL values were manually annotated following the protocol of Bahat and Irani (ICCP 2016) [22], which interactively selects distant sky or haze-dominated regions). To ensure a fair and rigorous comparison, the evaluation focuses exclusively on global AL estimation, as this represents a common functional requirement across all baseline methods. This consistency in parameter space ensures that the performance gains can be directly attributed to the accuracy of the underlying estimation logic. The quantitative and qualitative results of this comparison are detailed in Figure 9 and Figure 10.

Figure 9 presents a qualitative comparison of the atmospheric light (AL) estimation results across various methods, benchmarked against the ground truth for two representative test images (T1 and T2). To evaluate the algorithmic stability of each approach, we also provide AL estimates derived from localized image patches. The visual evidence confirms that our decoupled estimation framework yields AL values in significantly closer agreement with the ground truth in terms of both chromaticity (color) and intensity (magnitude). Furthermore, our approach demonstrates the highest degree of content invariance, as its local region estimates exhibit the least sensitivity to varying image textures and structures, thereby confirming its superior stability.

Complementing this qualitative analysis, Figure 10 provides a quantitative assessment of the L2-norm errors between the estimated and ground-truth AL for all evaluated algorithms across the 35 natural hazy images. Statistical analysis reveals that our method achieves the smallest median, mean, and variance of estimation errors. These metrics collectively validate the high precision and robust performance of our framework in accurately characterizing atmospheric light under diverse environmental conditions.

**Figure 11:**
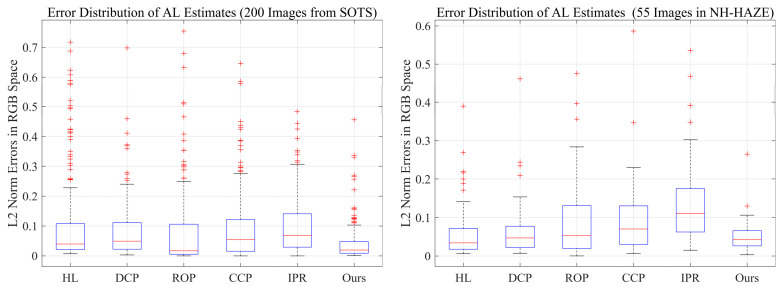
Boxplots of L2-norm errors of atmospheric light estimates for DCP, HL, IPR, ROP, CCP, and our method on the SOTS (200 images) (**left**) and NH-HAZE (55 images) (**right**) datasets.

To further evaluate the generalization of our AL estimation to public benchmarks, we extended the AL accuracy evaluation to 200 images from SOTS (selected for dense haze and infinite-depth regions) and all 55 images from NH-HAZE. Following the same manual annotation protocol of Bahat and Irani [22], ground-truth AL values were obtained for these images. We then computed the L2-norm error between the AL estimates of our method and the baseline methods against these ground-truth values. Figure 11 presents the error distributions as boxplots. Consistent with the results on the 35 natural hazy images, our method also achieves the lowest median error and the smallest interquartile range on both the SOTS and NH-HAZE datasets.

Beyond atmospheric light (AL) estimation accuracy, we performed an extensive evaluation of computational efficiency. The proposed framework comprises two primary stages: AL color estimation with a complexity of *O*(*NlogN*) and adaptive intensity estimation with a complexity of *O*(*N*), resulting in an overall computational complexity of *O*(*NlogN*). This efficiency profile is comparable to that of CCP [21] and represents a significant improvement over iterative or optimization-heavy paradigms.

While certain heuristics, such as DCP [4] and ROP [12], achieve linear complexity (*O*(*N*)), they often necessitate substantial trade-offs in estimation accuracy. Conversely, optimization-based frameworks (e.g., HL [6] and IPR [22]) are computationally expensive, often proving prohibitive for real-time processing. As evidenced by the execution times reported in Table 1, our method strikes an optimal balance between computational throughput and restoration performance. This positioning demonstrates that our approach is well-suited for practical, high-efficiency applications while consistently outperforming more complex, optimization-based benchmarks.

**Figure 12:**
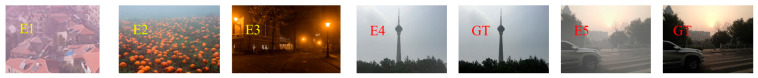
Natural hazy images E1–E3 and synthetic hazy images E4–E5 with ground truth (GT).

To evaluate the efficacy and generalizability of the proposed AL module, we integrate it into a diverse set of baseline dehazing frameworks. These benchmarks encompass four representative prior-based methods (DCP [4], ALSP [5], HL [6], and ROP [12]) as well as four state-of-the-art learning-based architectures (DehazeNet (DN) [1], MixDehazeNet (MDN) [13], RIDCP [14], and MSCNN [15]). The test images used in our experiments are shown in Figure 12.

Two distinct integration strategies were employed depending on the underlying architecture of each algorithm. For prior-based methods and certain modular networks, we directly replaced their internal atmospheric light estimation components with our decoupled module. For strictly end-to-end networks that do not explicitly output atmospheric light, our module is used as a preprocessing step to generate a pre-rectified hazy image as the refined input for these networks. In the former case, the integration can be considered plug-and-play, whereas in the latter case, we refer to it as preprocessing rectification to avoid implying an internal architectural modification. This distinction ensures a fair and accurate evaluation of the benefit provided by our decoupled AL estimation module. Specifically, before being processed by the end-to-end backbones, the original hazy images are jointly calibrated by our decoupled AL framework: the NPP corrects color casts, and the adaptive fusion mechanism balances exposure.

**Figure 13:**
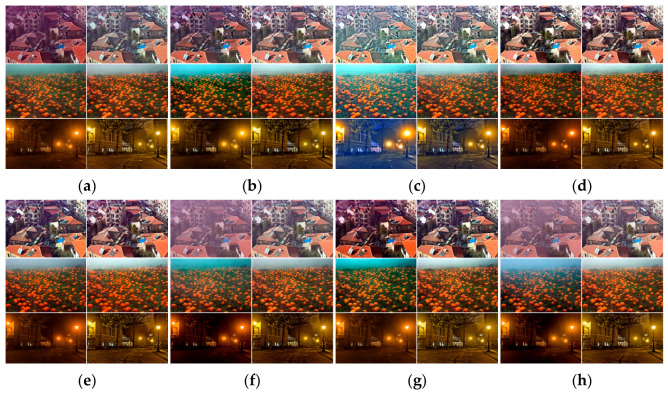
Side-by-side comparison of natural hazy images E1–E3. For each method, the left image shows the result using its own atmospheric light, while the right image shows the result using the atmospheric light estimated by our method. Subfigures (**a**–**h**) correspond to DCP, HL, ROP, ALSP, RIDCP, MSCNN, MDN, and DN, respectively.

**Figure 14:**
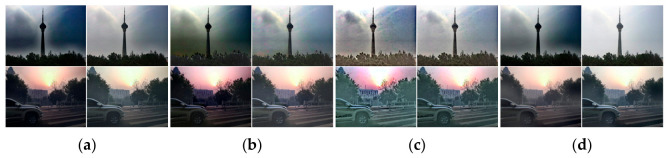

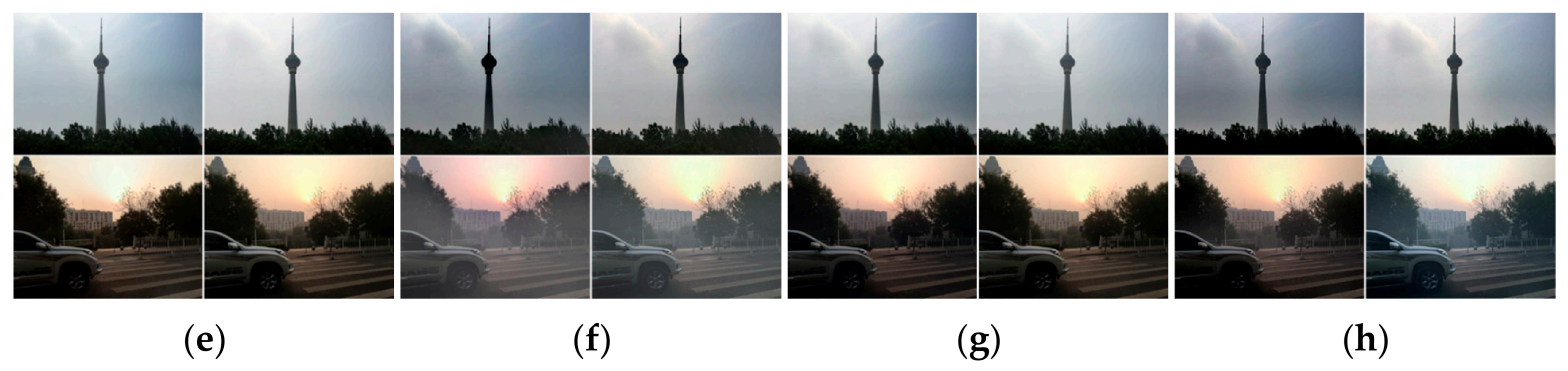
Side-by-side comparison of synthetic hazy images E4 and E5. For each method, the left image shows the result using its own atmospheric light, while the right image shows the result using the atmospheric light estimated by our method. Subfigures (**a**–**h**) correspond to DCP, HL, ROP, ALSP, RIDCP, MSCNN, MDN, and DN, respectively.

Figure 13 and Figure 14 illustrate the visual results for natural (E1–E3) and synthetic (E4–E5) hazy scenes, respectively. For each baseline method, the original output and the result enhanced by our AL-based rectification are displayed side-by-side to provide an intuitive demonstration of the consistent improvements in color fidelity and exposure stability.

A qualitative assessment of Figure 13 and Figure 14 confirms that replacing the baseline AL estimation with our decoupled module consistently improves performance across all tested paradigms. These side-by-side comparisons demonstrate a dual benefit: first, the neutral pixel prior (NPP) effectively neutralizes the color casts that often plague traditional methods; second, the adaptive fusion mechanism ensures optimal exposure even in regions with complex lighting.

Moreover, the synthetic results presented in Figure 14 highlight that our AL estimation aids in suppressing common dehazing artifacts, such as over-saturation in dense haze regions. By providing a more physically consistent AL estimate, our module enables the target dehazing methods to generate results that are not only visually pleasing but also more faithful to the ground truth. This consistency across diverse datasets underscores the robustness and versatility of our proposed approach.

To provide an objective assessment of the proposed method, we employed six widely-recognized dehazing benchmarks, categorized into no-reference and full-reference metrics. These include the fog aware density evaluator (FADE) [33], the apparent edge ratio (e), the normalized gradient of visible edges ($\bar{\gamma }$) [34], peak signal-to-noise ratio (PSNR), structural similarity index (SSIM), and CIEDE2000 [35].

For natural images where ground-truth references are unavailable, we use no-reference metrics (FADE, e, and $\bar{\gamma }$). In this context, a lower FADE score indicates more effective haze removal, while higher *e* and $\bar{\gamma }$ values reflect better restoration of scene details and edge contrast.

For synthetic datasets, we use full-reference metrics (PSNR, SSIM, and CIEDE2000) to quantify the deviation from the haze-free ground truth. Higher PSNR and SSIM values indicate better structural preservation and signal reconstruction, while a lower CIEDE2000 value denotes higher color accuracy and smaller perceptual color difference relative to the reference image.

Table 2 summarizes the quantitative results of the no-reference metrics on natural hazy images. The data show that replacing the native AL with our estimate consistently reduces FADE scores and increases e and $\bar{\gamma }$ values for most algorithms, with a few exceptions marked in red. This trend indicates clear improvements in haze removal, contrast, and visible details. Similarly, as reported in Table 3 for synthetic hazy images, integrating our AL generally yields higher PSNR and SSIM together with lower CIEDE2000 values (again with a few red-marked exceptions). These improvements confirm that the dehazed results achieve superior pixel-level fidelity, structural integrity, and color accuracy relative to the ground truth.

Although our AL module consistently improves the vast majority of metrics, a few isolated cases (marked in red in Table 2 and Table 3) show slightly worse values for some metrics. For instance, on images E1 and E5 with HL, HL includes an image enhancement step (gamma correction) that tends to over-enhance smooth or low-detail regions. Our adaptive intensity tuning suppresses such over-enhancement, leading to a slight decrease in the *e* value for E1 and in PSNR/SSIM for E5. For ROP, which uses per-channel transmission, our AL prevents over-dehazing in certain regions, which may marginally reduce the contrast enhancement in those areas (e.g., E1). For synthetic images E4 and E5, the ground truth itself contains a slight color cast; our AL corrects this cast, making the sky region lighter, which paradoxically increases the CIEDE2000 value for a few methods. These few exceptions do not undermine the overall improvement clearly shown by the full-reference metrics and visual quality.

To provide a detailed per-dataset comparison and statistical significance, we report the quantitative results on all five datasets separately. Table 4a lists the PSNR values (mean ± standard deviation) for each method under native AL and our AL, together with *p*-values from one-tailed paired *t*-tests. Table 4b,c present the corresponding SSIM and CIEDE2000 results, respectively. The *p*-values indicate whether our AL significantly outperforms native AL (for PSNR/SSIM, *p* < 0.05 means improvement; for CIEDE2000, *p* < 0.05 means significantly lower color difference).

From Table 4a–c, several observations can be made. First, our AL module consistently improves the performance of all eight baseline methods across all five datasets in terms of average PSNR and SSIM and reduces the average CIEDE2000. Second, the improvements are statistically significant for the vast majority of dataset-method pairs (*p* < 0.01 in most cases). Third, the gains are particularly pronounced on challenging datasets such as D-HAZE and NH-HAZE, which contain dense or non-uniform haze. These results fully confirm the robustness and generalization of our decoupled AL estimation.

**Figure 15:**
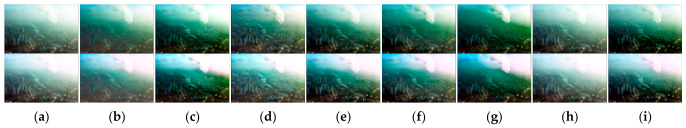
Failure case on a multi-color-cast scene. Our method cannot fully remove the color cast (e.g., cyan tint in the sky), illustrating the limitation of the single-chromatic-uniformity assumption. (**a**) Top: original hazy image; bottom: after our correction. (**b**–**i**) Comparison of eight methods (DCP, HL, ROP, ALSP, RIDCP, MSCNN, MDN, DN): native AL (**top**) vs. our AL (**bottom**).

Our method assumes that the atmospheric light color is globally uniform across the entire image. This assumption can be violated when different regions of the scene exhibit different atmospheric light colors, a condition we refer to as spatially varying chromaticity. A representative example is shown in Figure 15, where the hazy input contains a cyan-blue tint in the sky, a warm (yellowish) cast on the ground and buildings, and dense white haze around the chimney. These spatially varying atmospheric light colors cause our method to leave a slight residual color cast after dehazing (e.g., the sky still appears slightly cyan), revealing a limitation of the global uniformity assumption. No single atmospheric light color can completely eliminate all color casts in such scenes; moreover, correcting for one region may introduce a color shift or distortion in another. For instance, in panels (b)–(i) of Figure 15, the native AL of each method fails to remove the original color casts, while our AL corrects the dominant casts but still produces a subtle color shift in the dense white haze region (e.g., around the chimney). This issue has not received sufficient attention in most existing dehazing methods. In future work, we will focus on developing a spatially adaptive atmospheric light model that can handle locally varying chromaticity without introducing new artifacts.

## 5. Conclusions

This work addressed the pervasive issues of color cast and exposure imbalance in single image dehazing by proposing a novel decoupled atmospheric light (AL) estimation framework. By separating AL into independent color and intensity components, our method employs a neutral pixel prior (NPP) for robust spectral calibration and a depth-guided adaptive fusion mechanism for balanced exposure. This decoupling strategy provides a physically more grounded reformulation of the atmospheric scattering model (ASM). Extensive experiments on five public datasets (SOTS, D-HAZE, I-HAZE, NH-HAZE, O-HAZE) demonstrated that our approach not only achieves superior AL estimation accuracy but also consistently improves the color fidelity, exposure balance, and structural clarity of various ASM-based dehazing algorithms.

Despite these advances, the current method assumes globally uniform atmospheric light color, which may fail in scenes with spatially varying chromaticity (e.g., multi-color casts or dense white fog regions). As shown in the failure case (Figure 15), a single AL color vector cannot fully eliminate all color casts and may even introduce minor shifts in other areas. Future work will focus on three directions:

(1) Spatially adaptive AL estimation–relaxing the global uniformity assumption by estimating locally varying AL colors (e.g., via sparse reconstruction of the chromaticity field or depth guidance).
(2) Seamless integration with deep networks–embedding our decoupled physical prior as a lightweight, trainable module that can be inserted into existing deep dehazing architectures without retraining from scratch, enabling real-time adaptive correction.
(3) Joint optimization–combining the interpretability of our physical model with the representation power of deep learning to tackle more complex scenarios (e.g., night haze, mixed illumination).

## Figures and Tables

**Table 1:** Time Consumption of Our Method and Comparison Algorithms (s). (Bold indicates best per row).

Resolution	DCP	HL	IPR	CCP	ROP	Ours
500 × 500	**0.09**	3.76	10.41	0.12	0.11	0.19
700 × 700	**0.1** **2**	5.14	23.96	0.22	0.19	0.34
900 × 900	**0.** **2** **7**	6.68	40.33	0.48	0.37	0.51
1200 × 1200	**0.4** **0**	8.15	54.45	0.79	0.61	0.84

**Table 2:** FADE, e and $\bar{\gamma }$ scores: native (Na) vs. our (O) AL (Bold values are superior).

Metrics	Images	AL	DCP	HL	ROP	ALSP	RIDCP	MSCNN	MDN	DN
FADE	E1	Na	0.2934	0.3294	0.2887	0.2696	0.3280	0.6123	0.1882	0.6519
		O	**0.2675**	**0.2740**	**0.2760**	**0.1684**	**0.2327**	**0.3282**	**0.1668**	**0.3240**
	E2	Na	0.2383	0.1716	0.2877	0.1694	0.2308	0.2457	0.1731	0.2832
		O	**0.1496**	**0.1582**	**0.2461**	**0.1457**	**0.1621**	**0.1534**	**0.1684**	**0.1849**
	E3	Na	0.2942	0.2072	0.3106	0.2967	0.2685	0.2877	0.2856	0.2629
		O	**0.1821**	**0.1555**	**0.2458**	**0.1675**	**0.1751**	**0.1810**	**0.1852**	**0.1735**
*e*	E1	Na	1.2633	**1.2223**	1.1424	1.2787	1.2897	0.5781	1.3172	0.5129
		O	**1.4831**	1.1610	**1.1558**	**1.5978**	**1.4928**	**1.4963**	**1.4898**	**1.4143**
	E2	Na	0.2283	0.0795	0.1545	0.4437	0.2075	0.2606	0.1915	0.1445
		O	**0.3056**	**0.4219**	**0.1601**	**0.5428**	**0.3488**	**0.3744**	**0.2127**	**0.4440**
	E3	Na	0.0012	0.1274	0.2519	0.0883	0.0951	0.2346	0.0124	0.1202
		O	**0.1845**	**0.2243**	**0.2741**	**0.2492**	**0.2307**	**0.2764**	**0.1883**	**0.1833**
$\bar{\gamma }$	E1	Na	2.0382	2.9962	**5.8237**	4.0273	4.6227	1.4720	2.9981	1.4183
		O	**4.3914**	**3.2391**	5.3411	**5.1234**	**4.6491**	**4.0129**	**5.6227**	**3.9907**
	E2	Na	1.4368	1.8380	**2.5486**	2.9976	1.9178	1.3870	1.7349	1.2869
		O	**3.7298**	**3.2857**	2.4915	**4.0098**	**2.9322**	**3.5748**	**2.9470**	**2.9227**
	E3	Na	0.9996	1.3052	2.5020	1.0216	1.0683	0.9135	1.0014	1.0133
		O	**2.4829**	**2.4255**	**2.9156**	**2.6409**	**2.0878**	**2.5274**	**2.6638**	**2.5474**

**Table 3:** PSNR, SSIM, and CIEDE2000 scores: native (Na) vs. our (O) AL (Bold values are superior).

Metrics	Images	AL	DCP	HL	ROP	ALSP	RIDCP	MSCNN	MDN	DN
PSNR	E4	Na	13.17	13.52	14.49	14.78	30.58	18.69	26.08	17.11
		O	**17.21**	**17.70**	**18.15**	**16.76**	**32.90**	**22.59**	**28.05**	**18.19**
	E5	Na	20.65	21.58	15.72	19.69	27.66	15.21	34.32	22.88
		O	**22.92**	20.78	**16.98**	**20.92**	**28.77**	**15.93**	**36.16**	**24.40**
SSIM	E4	Na	0.6944	0.6246	0.6761	0.8318	0.9410	0.8878	0.9553	0.7595
		O	**0.7867**	**0.6980**	**0.7387**	**0.8512**	**0.9641**	**0.9067**	**0.9778**	**0.7967**
	E5	Na	0.7683	0.7580	0.3048	0.7160	0.9219	0.6646	0.9542	0.7247
		O	**0.7901**	0.7486	**0.4692**	**0.7756**	**0.9427**	**0.6815**	**0.9721**	**0.8232**
CIEDE 2000	E4	Na	9.60	10.59	13.24	16.86	**2.65**	8.89	3.32	10.98
		O	**9.05**	**9.76**	**6.94**	**8.15**	4.65	**4.08**	**3.09**	**3.86**
	E5	Na	25.18	27.67	21.79	**9.43**	3.55	14.84	2.94	**5.52**
		O	**9.82**	**12.21**	**14.75**	10.11	**2.74**	**13.23**	**2.17**	6.18

**Table 4:** (**a**) PSNR comparison between native AL and our AL (mean ± std) with statistical significance. (**b**) SSIM comparison between native AL and our AL (mean ± std) with statistical significance. (**c**) CIEDE2000 comparison between native AL and our AL (mean ± std) with statistical significance.

(**a**)									
Dataset	AL	DCP	HL	ROP	ALSP	RIDCP	MSCNN	MDN	DN
SOTS	Na	17.79 ± 3.46	18.60 ± 3.28	17.50 ± 1.88	20.09 ± 3.93	25.38 ± 2.29	21.87 ± 2.23	26.65 ± 2.89	23.73 ± 2.10
	O	19.24 ± 3.53	19.59 ± 2.65	19.62 ± 1.76	21.90 ± 3.51	26.01 ± 2.55	22.92 ± 2.10	27.59 ± 2.76	24.18 ± 2.02
	*p*-value	**	**	**	**	**	**	**	**
D-HAZE	Na	13.63 ± 1.98	11.66 ± 1.93	10.13 ± 2.44	12.10 ± 1.70	12.78 ± 2.41	11.45 ± 2.47	11.26 ± 2.60	10.99 ± 2.06
	O	15.59 ± 2.30	12.37 ± 2.05	11.73 ± 2.17	13.82 ± 1.76	14.23 ± 2.55	12.34 ± 2.61	13.25 ± 2.81	11.73 ± 2.11
	*p*-value	**	**	**	**	**	**	**	**
I-HAZE	N	16.28 ± 2.61	16.27 ± 2.44	16.28 ± 2.61	14.86 ± 2.32	17.02 ± 2.74	16.84 ± 2.24	16.39 ± 2.93	16.89 ± 2.77
	O	18.17 ± 2.04	17.12 ± 1.90	18.17 ± 2.04	17.45 ± 2.63	18.08 ± 1.95	17.68 ± 1.95	18.41 ± 2.85	17.76 ± 2.71
	*p*-value	**	**	**	**	**	**	**	**
NH-HAZE	Na	12.96 ± 1.81	12.03 ± 1.79	11.19 ± 1.85	12.66 ± 1.99	13.01 ± 2.20	12.06 ± 1.55	12.24 ± 1.84	12.28 ± 1.64
	O	14.01 ± 2.06	12.68 ± 1.87	13.64 ± 1.83	14.19 ± 2.09	13.99 ± 2.33	13.39 ± 1.48	13.81 ± 1.80	15.19 ± 2.09
	*p*-value	**	**	**	**	**	**	**	**
O-HAZE	Na	16.75 ± 2.90	14.82 ± 2.50	13.20 ± 3.57	16.67 ± 2.38	17.26 ± 2.68	16.41 ± 3.68	16.47 ± 3.41	15.67 ± 2.72
	O	17.38 ± 2.54	16.25 ± 2.40	14.95 ± 2.41	17.40 ± 2.36	18.35 ± 2.47	17.67 ± 2.97	17.90 ± 3.34	16.53 ± 2.80
	*p*-value	**	**	**	**	**	**	**	**
(**b**)									
Dataset	AL	DCP	HL	ROP	ALSP	RIDCP	MSCNN	MDN	DN
SOTS	N	0.887 ± 0.05	0.859 ± 0.07	0.740 ± 0.06	0.890 ± 0.05	0.922 ± 0.05	0.905 ± 0.05	0.932 ± 0.01	0.918 ± 0.05
	O	0.901 ± 0.04	0.871 ± 0.06	0.779 ± 0.05	0.912 ± 0.05	0.935 ± 0.04	0.917 ± 0.05	0.941 ± 0.01	0.929 ± 0.05
	*p*-value	**	**	**	**	**	**	**	**
D-HAZE	N	0.433 ± 0.11	0.456 ± 0.11	0.465 ± 0.12	0.439 ± 0.10	0.508 ± 0.11	0.415 ± 0.14	0.439 ± 0.10	0.412 ± 0.13
	O	0.531 ± 0.09	0.528 ± 0.11	0.486 ± 0.09	0.518 ± 0.08	0.534 ± 0.10	0.507 ± 0.11	0.518 ± 0.08	0.523 ± 0.11
	*p*-value	**	**	**	**	**	**	**	**
I-HAZE	Na	0.701 ± 0.09	0.773 ± 0.08	0.765 ± 0.07	0.738 ± 0.08	0.797 ± 0.06	0.766 ± 0.07	0.771 ± 0.10	0.749 ± 0.11
	O	0.798 ± 0.07	0.804 ± 0.07	0.772 ± 0.07	0.821 ± 0.05	0.833 ± 0.07	0.828 ± 0.07	0.856 ± 0.06	0.828 ± 0.09
	*p*-value	**	**	*	**	**	**	**	**
NH-HAZE	N	0.547 ± 0.09	0.579 ± 0.09	0.606 ± 0.07	0.597 ± 0.10	0.637 ± 0.08	0.530 ± 0.09	0.480 ± 0.08	0.519 ± 0.10
	O	0.656 ± 0.09	0.627 ± 0.08	0.673 ± 0.06	0.689 ± 0.09	0.692 ± 0.07	0.592 ± 0.08	0.569 ± 0.09	0.610 ± 0.10
	*p*-value	**	**	**	**	**	**	**	**
O-HAZE	Na	0.756 ± 0.09	0.744 ± 0.08	0.702 ± 0.09	0.761 ± 0.08	0.771 ± 0.04	0.753 ± 0.09	0.693 ± 0.13	0.760 ± 0.11
	O	0.812 ± 0.08	0.796 ± 0.07	0.736 ± 0.09	0.819 ± 0.07	0.824 ± 0.05	0.814 ± 0.07	0.772 ± 0.09	0.812 ± 0.10
	*p*-value	**	**	*	**	**	**	**	**
(**c**)									
Dataset	AL	DCP	HL	ROP	ALSP	RIDCP	MSCNN	MDN	DN
SOTS	Native	9.80 ± 3.98	9.16 ± 3.54	11.15 ± 2.62	8.41 ± 3.69	6.31 ± 2.04	7.91 ± 1.86	4.99 ± 1.26	6.96 ± 1.97
	Ours	8.28 ± 3.19	8.69 ± 2.67	8.75 ± 1.88	7.26 ± 2.70	6.07 ± 2.29	7.10 ± 1.62	4.08 ± 1.35	6.40 ± 2.05
	*p*-value	**	**	**	**	**	**	**	**
D-HAZE	Native	23.33 ± 4.41	25.81 ± 5.37	26.51 ± 6.85	23.90 ± 3.80	25.41 ± 4.59	23.81 ± 5.66	24.48 ± 6.63	23.81 ± 5.66
	Ours	20.98 ± 4.17	22.92 ± 5.15	22.56 ± 4.31	18.24 ± 3.63	23.38 ± 4.67	19.96 ± 5.33	23.62 ± 6.01	19.96 ± 5.33
	*p*-value	**	**	**	**	**	**	**	**
I-HAZE	Native	16.87 ± 4.38	13.54 ± 3.36	13.46 ± 3.58	15.55 ± 4.12	13.63 ± 3.50	12.32 ± 2.94	13.29 ± 4.28	13.02 ± 3.95
	Ours	13.92 ± 3.51	12.38 ± 2.04	10.74 ± 2.56	12.02 ± 3.81	12.09 ± 2.81	11.07 ± 2.72	10.06 ± 3.03	10.89 ± 3.52
	*p*-value	**	**	**	**	**	**	**	**
NH-HAZE	Native	21.84 ± 4.66	22.45 ± 4.51	22.05 ± 4.65	21.57 ± 4.66	19.21 ± 4.55	22.62 ± 3.71	21.56 ± 4.21	21.88 ± 4.10
	Ours	18.25 ± 4.23	20.50 ± 3.97	17.13 ± 3.30	16.40 ± 3.23	17.99 ± 4.36	18.97 ± 2.93	18.98 ± 4.10	16.78 ± 3.68
	*p*-value	**	**	**	**	**	**	**	**
O-HAZE	Native	15.96 ± 4.50	17.73 ± 4.54	20.32 ± 7.08	14.97 ± 4.16	15.25 ± 3.24	15.39 ± 4.22	15.68 ± 5.27	15.21 ± 5.00
	Ours	14.59 ± 4.14	14.92 ± 3.35	16.60 ± 3.97	12.13 ± 3.67	14.28 ± 3.47	14.76 ± 4.17	14.76 ± 5.54	14.36 ± 4.67
	*p*-value	**	**	**	**	**	**	**	**

Note: values are reported as mean ± standard deviation. *p*-values are from one-tailed paired *t*-tests comparing our AL versus native AL. For PSNR (Table 4a) and SSIM (Table 4b), a *p*-value < 0.05 indicates that our AL significantly outperforms native AL. For CIEDE2000 (Table 4c), a *p*-value < 0.05 indicates that our AL yields a significantly lower color difference (better color fidelity). Significance levels: ** *p* < 0.01.

## Data Availability

The data presented in this study are available in SOTS at https://utexas.app.box.com/s/uqvnbfo68kns1210z5k5j17cvazavcd1 (accessed on 10 March 2026), D-HAZY: https://www.kaggle.com/datasets/rajat95gupta/hazing-images-dataset-cvpr-2019 (accessed on 10 March 2026), O-HAZE: https://data.vision.ee.ethz.ch/cvl/ntire18/i-haze/ (accessed on 10 March 2026), I-HAZE: https://data.vision.ee.ethz.ch/cvl/ntire18/o-haze/ (accessed on 10 March 2026), Dense-Haze: https://data.vision.ee.ethz.ch/cvl/ntire20/nh-haze/ (accessed on 10 March 2026). Further inquiries can be directed to the corresponding author.
